# *DMD*‐related muscular dystrophy in Cameroon: Clinical and genetic profiles

**DOI:** 10.1002/mgg3.1362

**Published:** 2020-06-15

**Authors:** Edmond Wonkam‐Tingang, Séraphin Nguefack, Alina I. Esterhuizen, David Chelo, Ambroise Wonkam

**Affiliations:** ^1^ Division of Human Genetics Department of Pathology University of Cape Town Cape Town South Africa; ^2^ Department of Paediatrics Faculty of Medicine and Biomedical Sciences University of Yaoundé I Yaoundé Cameroon; ^3^ Paediatrics Unit Division of Paediatric Neurology Gynaeco‐Obstetric and Paediatric Hospital Yaoundé Cameroon; ^4^ National Health Laboratory Service Groote Schuur Hospital Cape Town South Africa; ^5^ Division of Paediatric Cardiology, Mother and Child Hospital Yaoundé Cameroon; ^6^ Department of Medicine University of Cape Town Cape Town South Africa

**Keywords:** Africa, Cameroon, clinical patterns, Duchenne muscular dystrophy, genetics

## Abstract

**Background:**

Most of the previous studies on Duchenne Muscular Dystrophy (DMD) were conducted in Caucasian, Asian, and Arab populations. Therefore, little is known about the features of this disease in Africans. In this study, we aimed to determine the clinical characteristics of DMD, and the common mutations associated with this condition in a group of Cameroonian patients.

**Methods:**

We recruited DMD patients and performed a general physical examination on each of them. Multiplex ligand‐dependant probe amplification was carried out to investigate exon deletions and duplications in the *DMD* gene (OMIM: 300377) of patients and their mothers.

**Results:**

A total of 17 male patients from 14 families were recruited, aged 14 ± 5.1 (8–23) years. The mean age at onset of symptoms was 4.6 ± 1.5 years, and the mean age at diagnosis was 12.1 ± 5.2 years. Proximal muscle weakness was noted in all patients and calf hypertrophy in the large majority of them (88.2%; 15/17). Flexion contractures were particularly frequent on the ankle (85.7%; 12/14). Wasting of shoulder girdle and thigh muscles was present in 50% (6/12) and 46.2% (6/13) of patients, respectively. No patient presented with hearing impairment. Deletions in *DMD* gene (OMIM: 300377) occurred in 45.5% of patients (5/11), while duplications were observed in 27.3% (3/11). Both mutation types were clustered between exons 45 and 50, and the proportion of de novo mutation was estimated at 18.2% (2/11).

**Conclusion:**

Despite the first symptoms of DMD occurring in infancy, the diagnosis is frequently made later in adolescence, indicating an underestimation of the number of cases of DMD in Cameroon. Future screening of deletions and duplications in patients from Cameroon should focus on the distal part of the gene.

## Graphical abstract

The study described a relatively late diagnosis of Duchenne Muscular Dystrophy (DMD) in Cameroon despite clinical signs and symptoms similar to those described in various other populations around the world. Exon deletions are the most frequent mutations associated with DMD in Cameroon and occur in almost half of patients. More awareness of this rare genetic disease is needed among the general population, policy‐makers, and clinicians in order to build capacity for the molecular diagnosis of genetic conditions in both Cameroon and the rest of Africa.

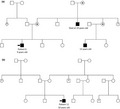

## 1 INTRODUCTION

Duchenne muscular dystrophy (DMD) is an X‐linked recessive disorder caused by mutations in the *DMD* gene (OMIM: 300,377), leading to the synthesis of a defective dystrophin protein (Suthar & Sankhyan, 2018). Dystrophin, in association with other muscle membrane proteins, makes a dystrophin‐associated protein complex that stabilizes the sarcolemmal membrane (Anthony et al., 2011; Rahimov & Kunkel, 2013; Suthar & Sankhyan, 2018). In the absence or reduction in dystrophin, the plasma membrane is fragile and predisposed to tearing and fragmentation during muscle fiber contraction (Rahimov & Kunkel, 2013; Suthar & Sankhyan, 2018). DMD has a world incidence of 1 in 3,500 live male births (Emery, 1991), and a global prevalence of 4.8 per 100,000 males (Mah et al., 2014). DMD prevalence in sub‐Saharan Africa has been estimated at 1/100,000 males (Ballo, Viljoen, & Beighton, 1994). DMD is the most common recessive X‐linked disease (Kapoor et al., 2012).

Most boys with DMD present symptoms between 3 and 5 years of age (Rao, Sindhav, & Mehta, 2014; Yiu & Kornberg, 2015). Delayed motor milestones, repeated falls, gait abnormalities, and difficulty climbing stairs are the most common symptoms at onset (Rao et al., 2014; Yiu & Kornberg, 2015). Subsequently, proximal and distal muscle weakness, calf hypertrophy, muscle atrophy, and contractures with orthopedic deformities occur (Swaminathan et al., 2009). In the early stages of DMD, the weakness of proximal muscles manifests by affected patients ‘climbing up their own bodies with their hands’ (Gower's sign) when rising from the floor to the standing position (Biggar, 2006). Serum creatine kinase (CK) levels and hepatic transaminases are usually elevated, reflecting active muscle degeneration (Yiu & Kornberg, 2015; Zatz et al., 1991). The severity of the disease is due to the eventual development of cardiac and respiratory abnormalities (Finsterer & Stöllberger, 2003; Kang, Kang, Sohn, Park, & Moon, 2006).

The *DMD* gene (OMIM: 300,377) is the largest known human gene, spanning 2.2 mbp and containing 79 exons (Aartsma‐Rus, Ginjaar, & Bushby, 2016; Kapoor et al., 2012). Deletions account for ~65% of *DMD* mutations that have been observed, while duplications have been measured in ~6%–10% of DMD patients. The remaining 30%–35% consist of small deletions, insertions, point mutations, or splicing mutations (Kapoor et al., 2012; Yiu & Kornberg, 2015). These mutations either disrupt the reading frame or generate a premature stop codon (Aartsma‐Rus et al., 2016). Deletions and duplications can occur anywhere in the gene, but they concentrate between exons 45–55 and exons 2–10, respectively (Aartsma‐Rus et al., 2016; Ankala et al., 2012). Mutations maintaining the reading frame of the *DMD* gene are often associated with a milder form of the disease called Becker muscular dystrophy (BMD; Yiu & Kornberg, 2015). Approximately one third of DMD cases are due to de novo mutations (Davie & Emery, 1978; Yiu & Kornberg, 2015).

Six loci for X‐linked hearing impairment (HI) have been described to date, including DFNX1 (Xq22.3), DFNX2 (Xq21.1), DFNX3 (Xp21.2), DFNX4 (Xp22.12), DFNX5 (Xq26.1), and DNFX6 (Xq22.3; Corvino et al., 2018). In 1994, Lalwani et al., (1994) reported a family with X‐linked nonsyndromic hearing impairment (NSHI) that mapped to region Xp21.2, which contains the *DMD* locus. A few years later, Pfister et al., (1999) reported a second family, from Turkey, with HI that mapped to the same locus. Interestingly, Raynor & Mulroy, (1997) demonstrated that *mdx* mice, an animal model for DMD, have an increased threshold for hearing when compared to normal mice. Additionally, the dystrophin protein was detected in the cochlea of guinea pigs and normal mice, but was absent in *mdx* mice (Dodson, Piper, Clarke, Quinlivan, & Dickson, 1995). Based on these results, these authors suggested that dystrophin might play a role in the hearing process, and recommended a systematic hearing screening of all DMD/BMD patients (Lalwani et al., 1994; Pfister et al., 1999).

Most of the previous studies on DMD were conducted in Caucasian, Asian, and Arab populations (Jumah et al., 2019; Saito et al., 2017; Yiu & Kornberg, 2015; Zhang et al., 2019). Therefore, little is known about the features and the burden of this condition in Africans. This study aimed to determine the clinical features of DMD and the most common mutations associated with this condition in Africans, and to assess a possible association with HI in a group of patients from Cameroon.

## 2 MATERIALS AND METHODS

### 2.1 Ethical compliance

This study was conducted in accordance with the Declaration of Helsinki. Ethical approval was granted by the Institutional Research Ethics Committee for Human Health of the Gynaeco‐Obstetric and Paediatric Hospital of Yaoundé, Cameroon (No. 723/CIERSH/DM/2015 and 137/CIERSH/DM/2018) and the University of Cape Town's Faculty of Health Sciences’ Human Research Ethics Committee (HREC 484/2019). Written and signed informed consent was obtained from all participants who were 21 years of age or older, and from parents or guardians in cases of minors, with verbal assent from participants, including permission to publish data.

### 2.2 Participants selection

We included patients admitted and treated for DMD, in the pediatric neurology unit of the Gynaeco‐Obstetric and Paediatric Hospital of Yaoundé, Cameroon, from January 2008 to May 2015. This cross‐sectional study had a retrospective component, from January 2008 to February 2015, mostly focused on participant recruitment and clinical data collection. It also included a prospective phase from February 2015 to May 2015, mainly dedicated to molecular analysis and clinical examination of patients who were still alive.

For all participants, detailed personal and family histories were collected. Medical records were reviewed by a medical team comprising of a general practitioner, a medical geneticist, a neuropediatrician and a cardiopediatrician when possible. Relevant data were collected, including three‐generation pedigrees, psychomotor development, age at onset, symptoms at onset, and age at diagnosis. A general physical examination was also performed.

### 2.3 Biochemical analyses

Biochemical measurements were obtained within 8 hr of drawing a peripheral blood sample in the biochemistry laboratory of the Centre Pasteur du Cameroon (CPC), Yaoundé, Cameroon. Participants were asked to refrain from excessive physical exercise for 48 hr prior to the blood sample collection. Analyses for CK, aspartate transaminase (AST), and alanine transaminase (ALT) were performed on the Vitros® autoanalyzer (Ortho‐clinical diagnostics, Johnson and Johnson) following the manufacturer's instructions and standard operating procedures. Enzymes activity levels were interpreted based on published standard age‐ and gender‐matched normal values (Kelishadi et al., 2012; Miri‐Dashe et al., 2014; Nicholson, Morgan, Meerkin, Strauss, & McLeod, 1985; Soldin, Murthy, Agarwalla, Ojeifo, & Chea, 1999).

### 2.4 Molecular analyses

Molecular analyses were performed in the National Health Laboratory Service, Groote Schuur Hospital and University of Cape Town, South Africa. DNA was isolated from peripheral blood using the salting out method as reported by Miller et al. (Miller, Dykes, & Polesky, 1988). Multiplex ligand‐dependant probe amplification (MLPA) was carried out using P034 and P035 probes from MRC Holland in both patients and parents. The 79 exons of the *DMD* gene were analyzed for the presence of exon deletions and/or duplications. The procedures and analyses were carried out as previously published (Murugan, Chandramohan, & Lakshmi, 2010; Sakthivel Murugan, Arthi, Thilothammal, & Lakshmi, 2013). The consequences of the identified mutations on the reading frame of the *DMD* gene were obtained through online search from Center for Human and Clinical Genetics, Leiden University Medical Center, (2004). The GenBank reference sequence used for the annotation of variants is NM_004006.2.

### 2.5 Definitions

The diagnosis of DMD was based on the presence of characteristic clinical signs (calf hypertrophy, muscle weakness, contractures, toe walking, frequent falls, difficulties climbing stairs, and Gowers’ sign), elevated serum muscle enzymes’ activities, and molecular analysis in some cases.

The hearing of our patients was assessed during clinical examination and was based on the ability of the patient to perceive speech with a normal voice. According to the recommendation number 02/1 bis of the Bureau International d’Audiophonologie (BIAP), Belgium (International Bureau for Audiophonologie 1997), an individual with a pure tone average of less than 41 dB can perceive speech with a normal voice. However, when the average tone is equal to or more than 41 dB, the person can only perceive speech if the voice is loud. The latter situation also corresponds to adult disabling HI, as stated by the World Health Organisation (WHO,^2020^). Based on this recommendation, only patients who could not perceive speech with a normal conversational voice were candidates for a formal audiology assessment.

A mother was considered as an obligate carrier in any of the following situations: she has one affected son as well as another affected male from her maternal lineage (Hutton & Thompson, 1976); she has two affected sons, or an affected son and a carrier daughter (Hutton & Thompson, 1976); on molecular analysis, both the mother and an affected son have a mutation in the *DMD* gene.

### 2.6 Data analysis

Descriptive statistic and nonparametric tests were used for comparisons, and *p* < 0.05 were considered statistically significant.

## 3 RESULTS

### 3.1 Participants’ demographics

**FIGURE 1:**
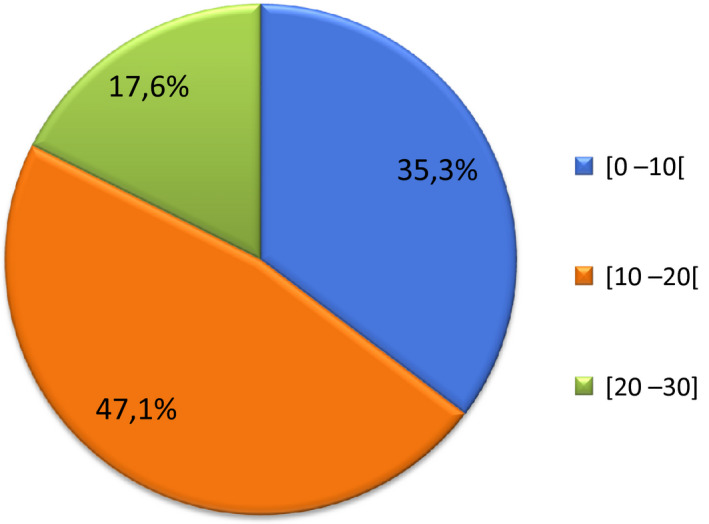
Distribution of our study population according to age range (years). *N* = 17 patients

A total of 17 patients from 14 families were investigated. All participants were male, aged 14 ± 5.1 years (range: 8–23 years). The majority of our patients (82.4%; 14/17) were aged <20 years (Figure 1). Nine patients were re‐examined by the research team, while the other patients’ clinical data were extracted from their pre‐existing medical records.

### 3.2 Medical history

#### 3.2.1 Psychomotor development

The mean age at onset of autonomous walking was 15.8 ± 4.1 months (range: 10–24 months). Six of our patients (35.3%; 6/17) had a history of delayed motor milestone development and 2 (11.8%; 2/17) had a history of delayed language acquisition.

#### 3.2.2 Onset and diagnosis

**FIGURE 2:**
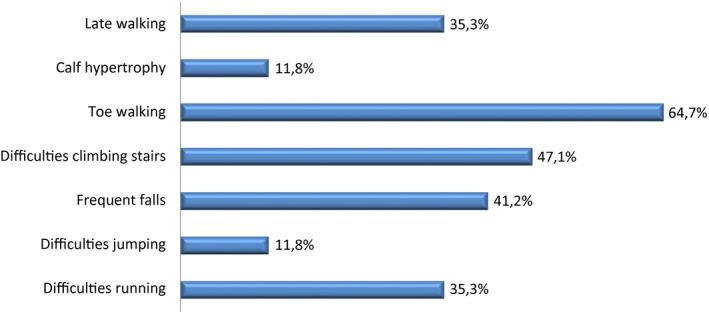
Clinical signs at onset of the disease. *N* = 17 patients

The mean age at onset of symptoms was 4.6 ± 1.5 years (range: 1–6 years; Table S1), and the mean age at diagnosis was 12.1 ± 5.2 years (range: 4–23 years; Table S2). Symptoms present at the onset of the disease are reported in Figure 2. The most common ones were toe walking, difficulties climbing stairs, and frequent falls, which were present in 64.7% (11/17), 47.1% (8/17), and 41.2% (7/17) of patients, respectively.

#### 3.2.3 Loss of ambulation

A proportion of patients (52.9%; 9/17) had expectedly lost ambulation at the time of the study. The mean age at loss of ambulation was 10.9 ± 2.1 years (range: 8–14 years).

### 3.3 Motor and orthopedic abnormalities

A wide range of motor abnormalities was observed in our patients and are summarized in Table 1. All patients who were still able to walk presented difficulties climbing stairs or running, frequent falls, toe walking, and inability to rise from the floor without using their arms (Gowers’ sign). All participants presented with proximal muscle weakness, particularly in the lower limbs. The large majority of participants had calf hypertrophy (88.2%; 15/17). Contracture in ankle plantar flexion (equine foot) was also observed in the majority of patients (85.7% 12/14). Muscle wasting of the shoulder girdle and thigh was noted in 50% (6/12) and 46.2% (6/13) of patients, respectively. Scoliosis was noted in 41.7% (5/12) of patients.

**TABLE 1:** Motor and orthopedic abnormalities

Clinical signs	*n*/*N*	Frequency (%)
Myalgia	5/11	45.5
Cramps	4/11	36.4
Difficulties climbing stairs	6/6	100
Difficulties running	7/7	100
Frequent falls	7/7	100
Toe walking	8/8	100
Gowers’ sign	8/8	100
Scoliosis	5/12	41.7
Contractures		
Elbow	5/12	41.7
Wrist	4/10	40
Fingers	2/9	22.2
Hips	6/11	54.5
Knees	7/13	53.8
Ankles (Equine foot)	12/14	85.7
Hypertrophy		
Tongue	2/11	18.2
Deltoid	4/12	33.3
Triceps	2/11	18.2
Quadriceps	2/10	20
Calf	15/17	88.2
wasting		
Neck muscles	3/10	30
Shoulder girdle muscles	5/12	41.7
Arm muscles	4/10	40
Forearm muscles	3/9	33.3
Pelvic girdle muscles	6/12	50
Tight muscles	6/13	46.2
Leg muscles	4/10	40
Neck weakness		
Neck flexors	7/9	77.8
Neck extensors	6/9	66.7
Sternocleidomastoid	6/9	66.7
Upper limbs weakness		
Deltoid	9/9	100
Pectorals	9/9	100
Biceps	10/12	83.3
Triceps	8/10	80
Wrist flexors	5/9	55.6
Wrist extensors	5/9	55.6
Lower limbs weakness		
Iliopsoas	14/14	100
Gluteus maximus	14/14	100
Hip adductors	14/14	100
Hip abductors	14/14	100
Quadriceps	14/14	100
Hamstrings	14/14	100
Tibialis anterior	7/10	70
Gastrocnemius	7/10	70

Note

*N*, number of patients in whom the clinical sign was checked; *n*, number of patients in whom the clinical sign was found.

### 3.4 Other clinical abnormalities

None of our patients presented with HI. They were all able to perceive speech with normal voice, meaning that their pure tone averages were less than 41 dB. Two patients (20 and 21 years old) had clinical signs of heart failure (Table 2), and their echocardiograms further highlighted a dilated cardiomyopathy. Symptoms suggestive of obstructive sleep apnea and nocturnal hypoventilation (fatigue, morning headaches, daytime sleepiness, snoring, and abrupt awakening accompanied by choking) were present in two patients (Table 2), including one (the 23‐year‐old patient) who presented with respiratory insufficiency. Three patients had at least two gastrointestinal symptoms, and two others had urological symptoms (Table 2).

**TABLE 2:** Other clinical findings

Clinical signs	*n*/*N*	Frequency (%)
Signs of heart failure		
Dyspnea	3/10	30
Orthopnea	2/10	20
Displaced apex beat	2/10	20
Tachycardia	3/10	30
Third heart sound	2/9	22.2
Gallop rhythm	2/9	22.2
Heart murmur	2/9	22.2
Abnormal pulse	1/9	11.1
Symptoms of sleep‐disordered breathing		
Morning headaches	2/9	22.2
Daytime sleepiness	2/9	22.2
Fatigue	2/9	22.2
Snoring	2/9	22.2
Abrupt awakenings and choking	2/9	22.2
Trouble concentrating	1/9	11.1
Night‐time sweating	1/9	11.1
Gastrointestinal symptoms		
Abdominal distention	3/9	33.3
Constipation	3/9	33.3
Dysphagia	1/9	11.1
Regurgitation	1/9	11.1
Urological symptoms		
Urinary frequency	2/9	22.2
Urinary urgency	1/9	11.1
Nocturia	2/9	22.2
Enuresis	3/9	33.3

Note

*N*, number of patients in whom the clinical sign was checked; *n*, number of patients in whom the clinical sign was found.

### 3.5 Enzymes

Mean serum enzymes’ activities are reported in Table 3. CK levels were above normal range values for age in all patients (*N* = 17) and screened mothers (*N* = 4). All patients (*N* = 17) had CK levels 10 times higher than the maximal limits for age, including four patients (23.5%; 3/17) with CK activities 100 times higher than the upper normal limit. Serum ALT activities were raised in all patients (*N* = 15), and AST activities were raised in almost all patients (93.3%; 14/15).

**TABLE 3:** Serum muscle enzymes levels

Enzymes	*N*	Mean values	Min.	Max.	Normal ranges
CK (U/L)	17	8,171.2 ± 7,545.3	837	31,872	30–150
AST (U/L)	15	183.4 ± 133.0	45	558	8–50
ALT (U/L)	15	243.1 ± 163.7	51	606	4–49
Maternal CK (U/L)	4	434.5 ± 450.6	155	1,104	20–140

Note

ALT, alanine transaminase; AST, aspartate transaminase; CK, creatine kinase; Min., minimal value; Max., maximal value; *N*, number of participants in whom serum enzymes activities have been measured.

### 3.6 Genetic profile

#### 3.6.1 Inheritance patterns

In five families (5/14; 35.7%; Figure 3, Figures S1–S12), the pedigrees were compatible with an X‐linked recessive inheritance pattern, with at least two affected boys. While in the remaining nine families (9/14; 64.3%), the patient was the only affected person, suggesting a de novo mutational event (Figure 3; Figures S1–S12). None of our patients were the offspring of a consanguineous relationship.

**FIGURE 3:**
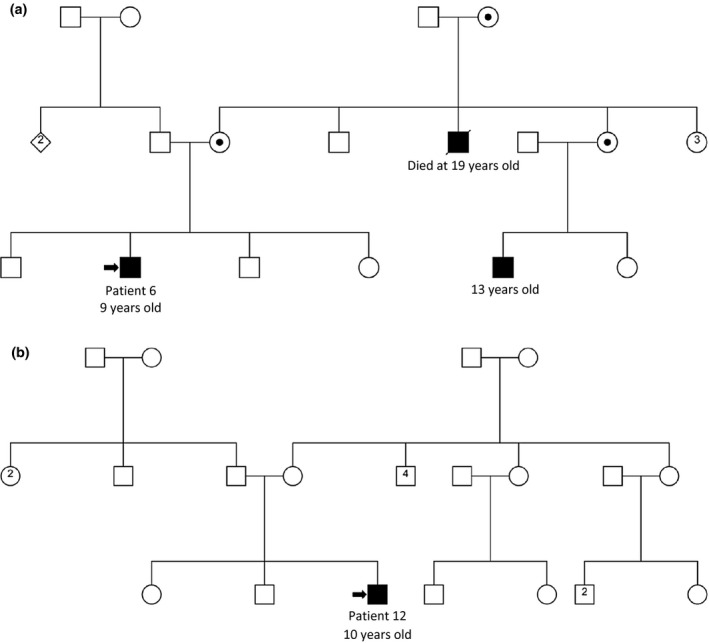
Inheritance pattern of DMD in our cohort. (a) Pedigree of a multiplex family (family 5), suggestive of X‐linked inheritance. The proband here has a cousin who exhibits clinical signs of DMD, and a deceased uncle who has presented the same clinical signs. (b) Pedigree of a family (family 9) in which the disease seems to be of sporadic occurrence. Arrows indicate the proband

#### 3.6.2 Mutations associated with DMD in our cohort

**FIGURE 4:**
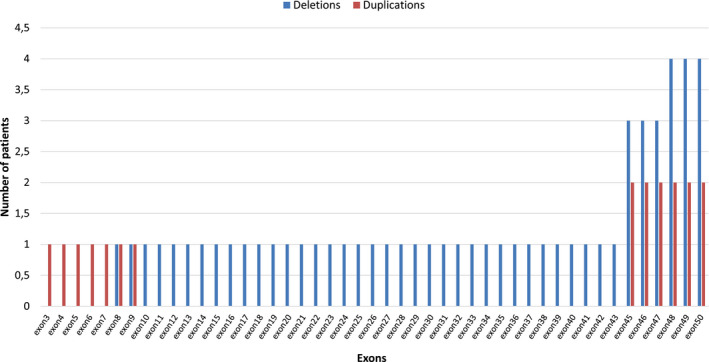
Distribution of the nature of mutations in patients according to affected exons

MLPA was performed in 11 patients, and four mothers. Exon deletions were found in five patients (45.5%; 5/11), and duplications found in three (27.3%; 3/11). In the remaining three patients, the MLPA did not reveal any mutation. Of the five patients in whom deletions occurred, three had deletions of six exons (exons 45–50), one patient displayed deletions of three exons (exons 48–50), and the remaining patient had deletions of 36 consecutive exons (exons 8–43). With regards to duplications, two patients had duplications of six exons (exons 45–50), while the last patient had duplications of seven exons (exons 3–9). The impact of the identified mutations on the reading frame of the *DMD* gene is reported in Table 4. Most commonly affected exons for both deletions and duplications were exons 45–50 (Figure 4). No correlation was found between mutation location and motor impairments.

**TABLE 4:** List of mutations identified in our cohort

Mutation	HGVS nomenclature^a^	Translational effect^a^	ISCN (2013) nomenclature^b^	*n*
Deletion exons 45–50	c.6439‐?_7309+?del	Frame‐shift	rsa Xp21.2(31,819,975‐31,968,514)x0	03
Deletion exons 48–50	c.6913‐?_7309+?del	Frame‐shift	rsa Xp21.2(31,819,975–31,875,376)x0	01
Deletion exons 8–43	c.650‐?_6290+?del	Frame‐shift	rsa Xp21.2(32,287,529‐32,699,293)x0	01
Duplication exons 45–50	c.6439‐?_7309+?dup	Frame‐shift	rsa Xp21.2(31,819,975–31,968,514)x2	02
Duplication exons 3–9	c.94‐?_960+?dup	In‐frame	rsa Xp21.2(32,697,870‐32,849,820)x2	01

Note

?, The actual breakpoint is unknown; HGVS, Human Genome Variation Society; ISCN, International System for Human Cytogenetic Nomenclature; *n*, number of patients carrying the mutation; rsa, region‐specific assay.

^a^ Data obtained through online search from Center for Human and Clinical Genetics, Leiden University Medical Center, (2004, RefSeq: NM_004006.2.

^b^ Corresponding genomic coordinates were retrieved from Center for Human and Clinical Genetics, Leiden University Medical Center, (2004, build GRCh38/hg38.

#### 3.6.3 Carrier status

Five mothers were from multiplex families and were thus designated obligate carriers. Molecular testing was performed for four mothers. Duplications were found in two mothers (including one from a multiplex family), while, in the remaining two, deletions identified in their respective sons were not found in their own *DMD* genes, suggesting that these were de novo events.

Combining pedigree analysis and molecular testing, we established that of the 14 mothers included in our cohort, 6 (related to 9 of the patients) were carriers, 2 (2 patients) were noncarriers, and a definite status could not be defined for the remaining 6 (6 patients). The proportion of de novo mutations in this study population was therefore estimated at 25% (2/8) in families with positive molecular analysis, corresponding to 18.2% (2/11) among all patients.

## 4 DISCUSSION

This study is one of the rare reports from Central Africa that describes clinical and genetic profile of *DMD*‐related Muscular Dystrophy. Despites the molecular testing been performed in relatively limited number of patients (11/17), the data provide the much needed insight into the features of this condition among Black Africans.

The mean age at onset of the first symptoms at 4.6 years (Table S1) was similar to that previously described in India, where the mean at onset of the disease was found to be 4.1 years (Rao et al., 2014). This is, however, higher than the 2.4 years reported by Alvarez Leal et al. (Alvarez Leal et al., 1994) in Mexico. Similar symptoms at the onset of the disease, including delayed motor milestone, toe walking, frequent falls, late walking, and difficulties running and climbing stairs, were reported previously in the United States of America (USA), Italy, and Kuwait (Ciafaloni et al., 2009; D’Amico et al., 2017; Mohammed et al., 2018). In this study, despite the occurrence of symptoms in infancy among patients, the clinical diagnosis of DMD was only established at a later age (12.1 ± 5.2 years), aided by the establishment of a medical genetic service in Cameroon (Wonkam et al., 2011). This late diagnosis (Table S2) suggests a possible underestimation of the actual number of cases of DMD in Cameroon. It also further highlights the need to raise awareness among clinicians, in order to improve early referral, diagnosis, and management of the condition, and the need to build capacity for the molecular diagnosis of DMD and other genetic conditions, in general, in Cameroon.

The distribution of muscle weakness, muscle wasting, hypertrophy, and contractures noted in the study participants is similar to that described in multiple populations from India, Korea, and Tunisia (Dey et al., 2015; Hamida, Miladi, Turki, & Zaiem, 1992; Lee et al., 2012; Swaminathan et al., 2009). CK levels are comparable to those reported in the literature (Yiu & Kornberg, 2015). The variable expression in terms of age of onset and clinical severity was observed, even between patients from the same family has also been reported before (Brooke et al., 1983; Hamida et al., 1992; Swaminathan et al., 2009). Spinal deformity is common in muscular dystrophies, usually occurring after the loss of walking ability (Fürderer, Hopf, Zöllner, & Eysel, 2000). The mean age at loss of ambulation in our study population was 10.9 years, which is in line with findings from studies conducted in other populations, including France (Desguerre et al., 2009). Similar to the findings described in this group of Cameroonian patients with DMD, heart failure and respiratory insufficiency were shown in previous reports to occur at an advanced stage of the disease (Melacini et al., 1996; Schram et al., 2013).

Like the present study, DMD and BMD have been described in a few African countries. A group of 11 patients from Nigeria (a West African country) were reported some decades ago (Dada & Elliott, 1967). Clinical signs described in this group of patients were similar to those reported in our study, including frequent falls, Gowers’ sign, calf hypertrophy, muscle weakness, and contracture. However, the mean age at onset of symptoms in that Nigerian cohort was 7.7 years, which is higher than the 4.6 years reported in the present study. In 2014, a cohort of six patients from Rwanda (an East‐African country) presenting with the DMD phenotype was described (Uwineza et al., 2014). The clinical and biological findings (CK levels) were similar to those described in our cohort. Like in the present study, they used MPLA to screen their patients for mutations in the *DMD* gene, with a detection rate of 66.7% (4/6) compared to 72.7% (8/11) in our study. These findings highlighted the efficacy of MLPA in detection copy number variations in the *DMD* gene. A 9‐year‐old boy presenting typical clinical signs of DMD was described in Tanzania, as well as a familial case of BMD (Dekker et al., 2019; Mgone & Kimati, 1981). A large cohort of DMD and BMD patients (*n* = 128) from Southern Africa was reported, with a prevalence of deletion of 46%, which is similar to the 45.5% reported in the present study (Ballo et al., 1994).

Previous studies have demonstrated a significant sensorineural hearing loss associated with muscular dystrophy in the mdx mouse model (Raynor & Mulroy, 1997). Mutations in families with DFNX3 deafness might occur in a novel gene located within the Xp21.2 locus (Lalwani et al., 1994), however, among families previously reported as having segregating HI that mapped to the *DMD* locus (Xp21.2), none presented with any of the typical clinical signs of DMD (Lalwani et al., 1994; Pfister et al., 1999). Additionally, although HI was found to be associated with some muscular dystrophies, including facioscapulohumeral muscular dystrophy and limb‐girdle muscular dystrophy (Brouwer et al., 1991; Mah & Chen, 2018; McDonald, Stajich, Blach, Ashley‐Koch, & Hauser, 2012), it was not clinically proven in humans affected with DMD (Allen, 1973). This study concurs with these findings.

This study confirms that deletions in the *DMD* gene are the most frequent mutations associated with the disease in Cameroon. The prevalence of deletions in the *DMD* gene varies greatly across populations, that is, 37%, 64%, and 86% among Israeli, Caucasians, and Arabs, respectively (Bresolin et al., 1994; Haider, Bastaki, Habib, & Moosa, 1998; Shomrat, Gluck, Legum, & Shiloh, 1994). Previous studies have demonstrated that 50% to 82% of deletions occur within the distal region of the *DMD* gene, including exons 44–55 (Haider et al., 1998; Shomrat et al., 1994; Thakur, Abeysekera, Wanigasinghe, & Dissanayake, 2019; Vengalil et al., 2017); this is in line with the findings of the present study, as 80% (4/5) of our patients had deletions between exons 45 and 50. The frequency of duplications in our cohort (27.3%; 3/11) was higher than that of populations from the Netherlands, China, and India, in which duplications were found in 7%, 8.4%, and 9% of DMD patients, respectively (Vengalil et al., 2017; White et al., 2006; Xu et al., 2018).

The exonic distribution of duplications in our patients also differed from previous reports. In two of our three patients (66.7%) with duplications, mutations occurred between exons 45 and 50, while previously published papers reported that duplications are most commonly clustered toward the 5’ end of the gene, between exons 2 and 25 (Ma et al., 2018; Okubo et al., 2017; Vengalil et al., 2017; White et al., 2006). In three of our patients (27.3%; 3/11) in whom MLPA was performed, no mutation was identified, suggesting the occurrence of small mutations that still need to be investigated (including insertions, small deletions, single nucleotide variants, and splice site mutations). These small mutations, which are ideally screened through sequencing techniques, were found in 19%, 22%, and 34.2% of DMD patients from China, Japan, and Spain, respectively (Ma et al., 2018; Okubo et al., 2017; Vieitez et al., 2017). Point mutations were described in two Western‐African families from Mali (Meilleur et al., 2011).

Seven patients presented an out‐of‐frame mutation, and their clinical profiles were compatible with a severe form of DMD. One patient presented an in‐frame duplication of exons 3–9. Although this 10‐year‐old patient has not yet lost ambulation, he could not walk without support or crutches, and his phenotype was too severe to be associated with BMD. Exceptions to the reading‐frame rule have also been described in the Rwandan cohort, where two patients with the DMD phenotype presented an in‐frame deletion (Uwineza et al., 2014), and in Canada where in‐frame duplications were reported in two DMD patients (Hu, Ray, Murphy, Thompson, & Worton, 1990). Three of the eight patients who had not yet lost ambulation did not benefit from a molecular screening. Two of them were 8 years old, and the last one was 12 years old. Although their phenotypes were compatible with the severe form of the DMD, the possibility of BMD could not be excluded. BMD is less frequent than DMD, with a prevalence in sub‐Saharan Africa estimated at 1/755,000 males (Ballo et al., 1994). BMD patients have a milder phenotype, with a lower progression as compared to DMD patients. The first symptoms appear around 12 years of age, and the loss of ambulation never occurs before 16 years old (Uwineza et al., 2014). BMD is most common due to in‐frame mutations in the *DMD* gene (Aartsma‐Rus, Van Deutekom, Fokkema, Van Ommen, & Den Dunnen, 2006). An Eastern‐African family from Tanzania presenting BMD was reported, with an in‐frame deletion of exons 45–48 (Dekker et al., 2019).

The proportion of de novo mutations in the present study was estimated at 18.2%, which is lower than the theoretical estimate of one third predicted by Haldane's rule (Haldane, 2004), probably due to the relatively modest sample size. The frequency of new mutants among patients with DMD ranges from 16.4% to 39.5% in Caucasians and Asians (Bucher, Ionasescu, & Hanson, 1980; Kong et al., 2019; Patiño, Narbona, & García‐Delgado, 1995), and is up to 62.2% in Latin Americans (Alcántara et al., 1999). In four of our carrier mothers, CK levels were above the normal range values, which means that the assay of CK activity may be a reliable and cost‐effective criterion to determine carrier status (Fitzsimmons et al., 1980; Hutton & Thompson, 1976), especially in sub‐Saharan African settings where molecular diagnosis facilities are not always available. The determination of carrier status is critical for the genetic counseling (Lane, Robinow, & Roses, 1983). Women at risk of being carriers in families included in our study were offered a retrospective molecular screening and were informed of the existence of antenatal molecular diagnosis, which is now possible in Cameroon (Wonkam et al., 2011).

Limitations of this study include the clinical assessment of hearing of our patients as it was solely based on conversational voice. As a result, patients with mild/moderate HI could have been missed. Additionally, retrospective clinical data extraction from medical records was incomplete due to poor medical archiving. In addition, intellectual disability was not formally assessed; intellectual disability has been estimated to occur in ~32% of DMD patients (Dubowitz, 1965). Lastly, molecular analysis was not performed for all patients. Targeted sequencing of the whole *DMD* gene should be performed in future to identify small mutations associated with DMD in the Cameroonian population, which were not detected by MLPA. Nevertheless, the study had some benefits for these families with a rare disease that deserves to be the object of more attention in Africa. Affected families were encouraged to form support groups to raise awareness among the general population and policy‐makers, to share their experience of the disease, and to help each other face their challenges. Further studies are needed to estimate the national prevalence and incidence of DMD in Cameroon.

## 5 CONCLUSION

The study described a relatively late diagnosis of DMD in Cameroon, despite clinical signs and symptoms similar to those described in various other populations around the world. HI was not associated with DMD in our study population. Exon deletions are the most frequent mutations associated with DMD in Cameroon and occur in almost half of patients. The frequency of duplications in the Cameroonian population seems to be higher than that reported in other populations. We suggest that future screening of deletions and duplications in patients from Cameroon, when analysis of all the 79 exons of the *DMD* gene is not feasible, should be focused on the distal part of the gene between exons 45 and 50. More awareness of this rare genetic disease is needed among the general population, policy‐makers, and clinicians in order to build capacity for the molecular diagnosis of genetic conditions in both Cameroon and the rest of Africa.

## CONFLICT OF INTEREST

We declare that no competing interests exist.

## AUTHORS’ CONTRIBUTIONS

A.W. and S.N. conceived the project and E.W.T. developed the protocol. E.W.T., S.N., D.C., and A.W. took medical histories and performed clinical examinations of patients. A.I.E. performed molecular analyses. E.W.T. compiled all the results and issued the first draft of the manuscript. S.N., A.I.E., D.C., and A.W. critically revised successive drafts of the manuscript. S.N. and A.W. supervised the project and compiled the revisions. All authors agree to the final version of the manuscript.

## Supporting information

Supplementary Material

## Data Availability

The data that support the findings of this study are available from the corresponding author upon reasonable request.
