# Supplementary material for: DMD‐related muscular dystrophy in Cameroon: Clinical and genetic profiles
Source: Mol Genet Genomic Med. 2020 Jun 15;8(8):e1362. doi: 10.1002/mgg3.1362 (PMC7434738; doi:10.1002/mgg3.1362)
Supplement: Supplementary file 1 — Supplementary Material [file MGG3-8-e1362-s001.pdf]

SUPPORTING INFORMATION

Duchenne Muscular Dystrophy in Cameroon: Clinical and Genetic Profiles

Edmond Wonkam-Tingang, Séraphin Nguefack, Alina I. Esterhuizen, David Chelo, Elie Mbonda and Ambroise Wonkam

Content:

Table S1: Age ranges at onset of the disease 2
Table S2: Age ranges at diagnosis 2
Figure S1: Pedigree of family 1 2
Figure S2: Pedigree of family 2 3
Figure S3: Pedigree of family 3 4
Figure S4: Pedigree of family 4 5
Figure S5: Pedigree of family 6 6
Figure S6: Pedigree of family 7 6
Figure S7: Pedigree of family 8 7
Figure S8: Pedigree of family 10 8
Figure S9: Pedigree of family 11 9
Figure S10: Pedigree of family 12 10
Figure S11: Pedigree of family 13 11
Figure S12: Pedigree of family 14 12

Table S1: Age ranges at onset of the disease

Age ranges (years)	n	Frequency (%)
[0 – 5[	9	52.9
[5 – 10[	8	47.1
Total	17	100

n, number of patients

Table S2: Age ranges at diagnosis

Age ranges (years)	n	Frequency (%)
[0 – 10[	6	35.3
[10 – 20[	9	52.9
[20 – 30]	2	11.8
Total	17	100

n, number of patients

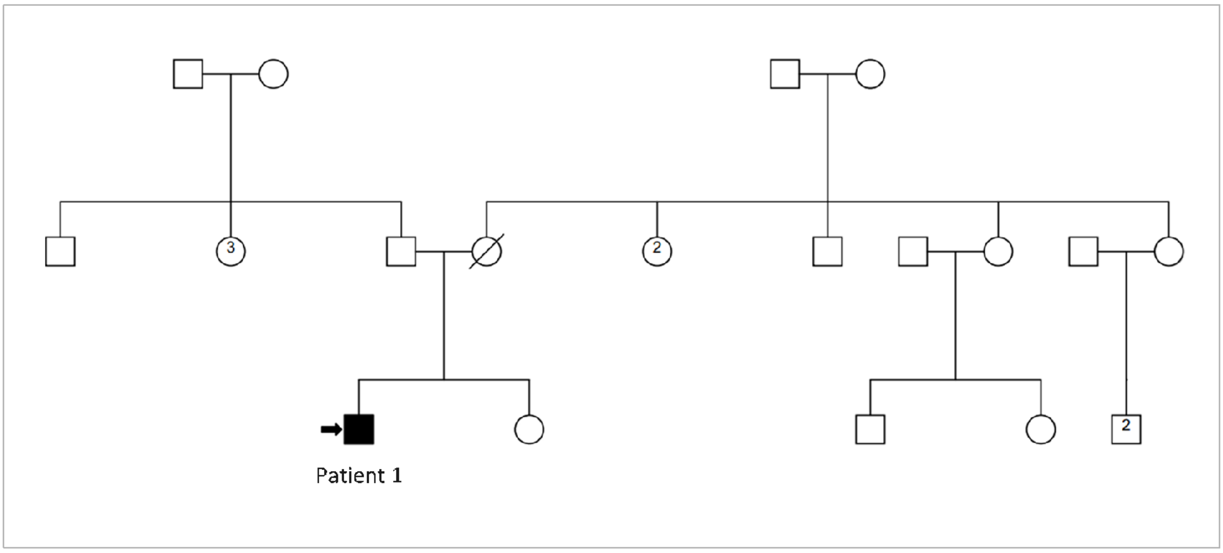

Figure S1: Pedigree of family 1

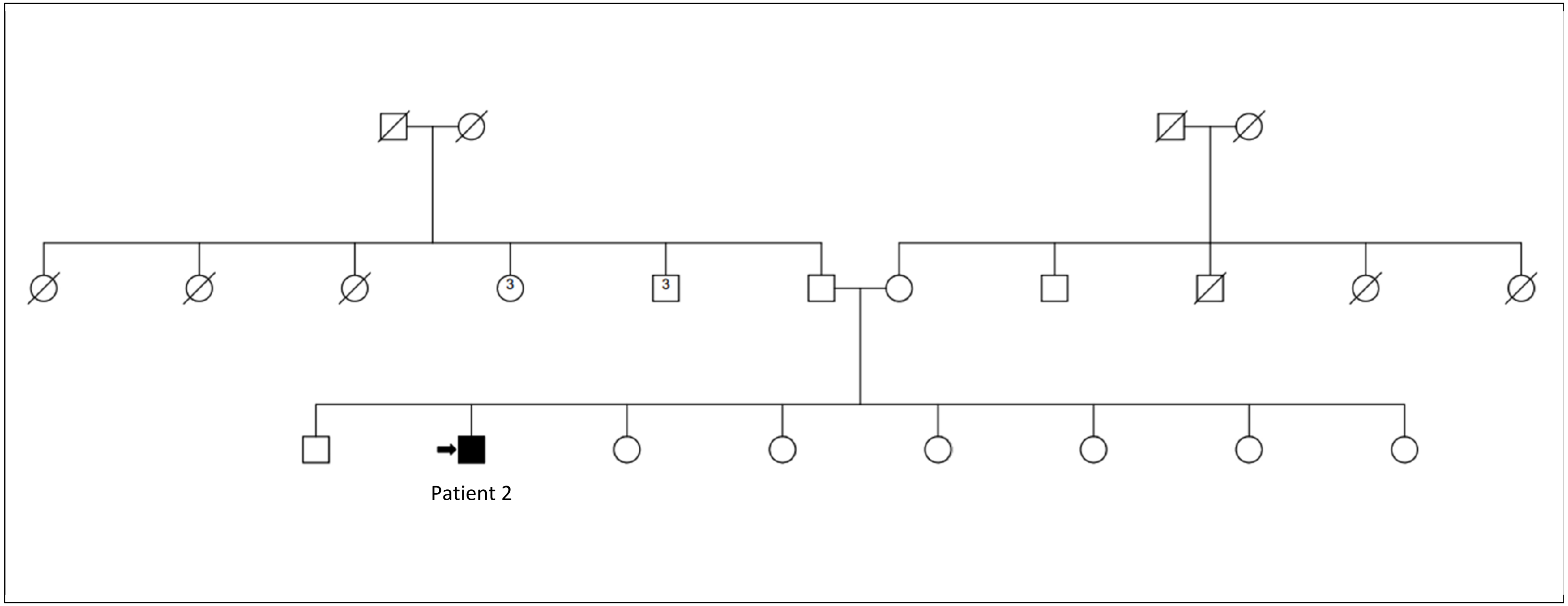

Figure S2: Pedigree of family 2

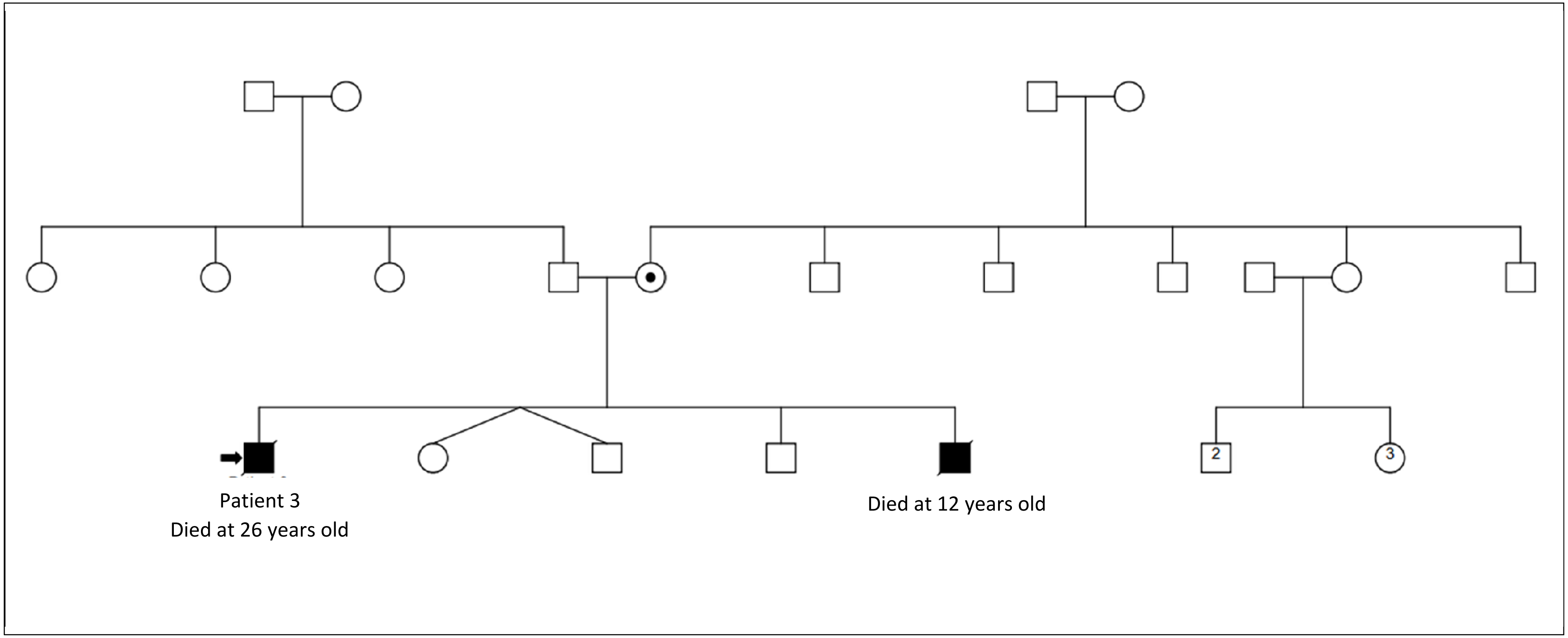

Figure S3: Pedigree of family 3

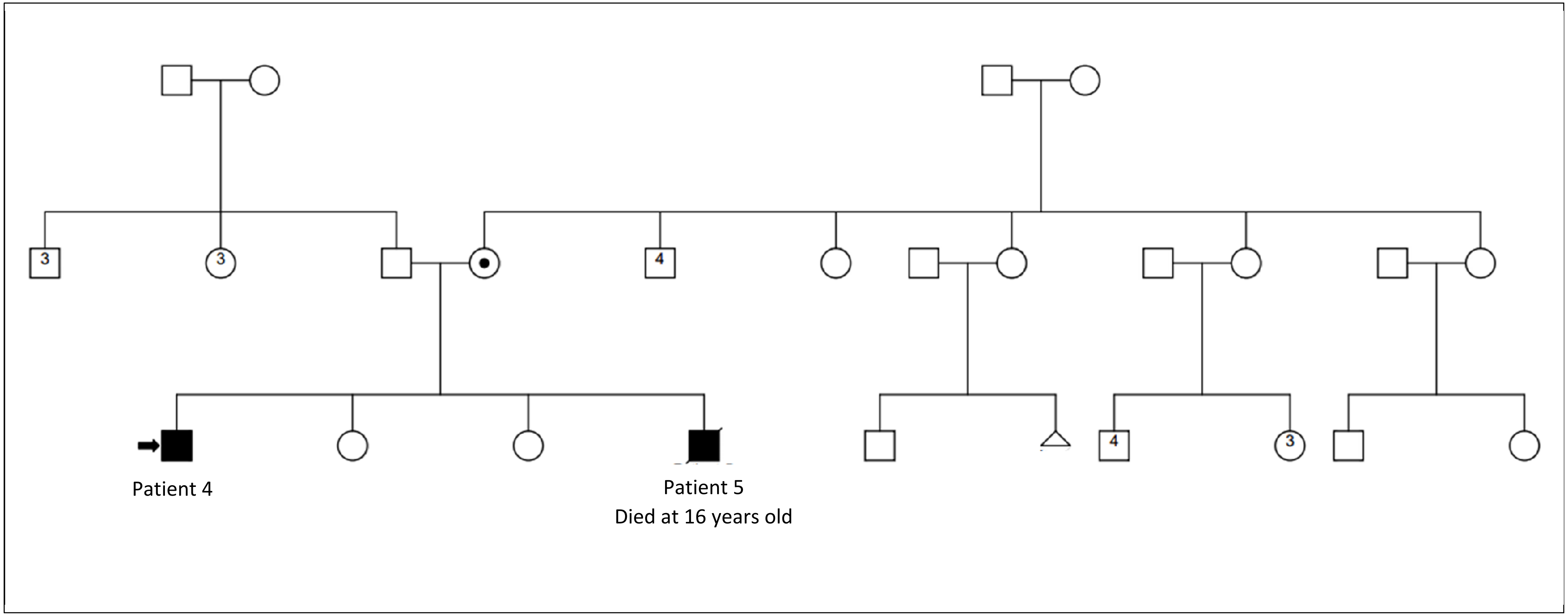

Figure S4: Pedigree of family 4

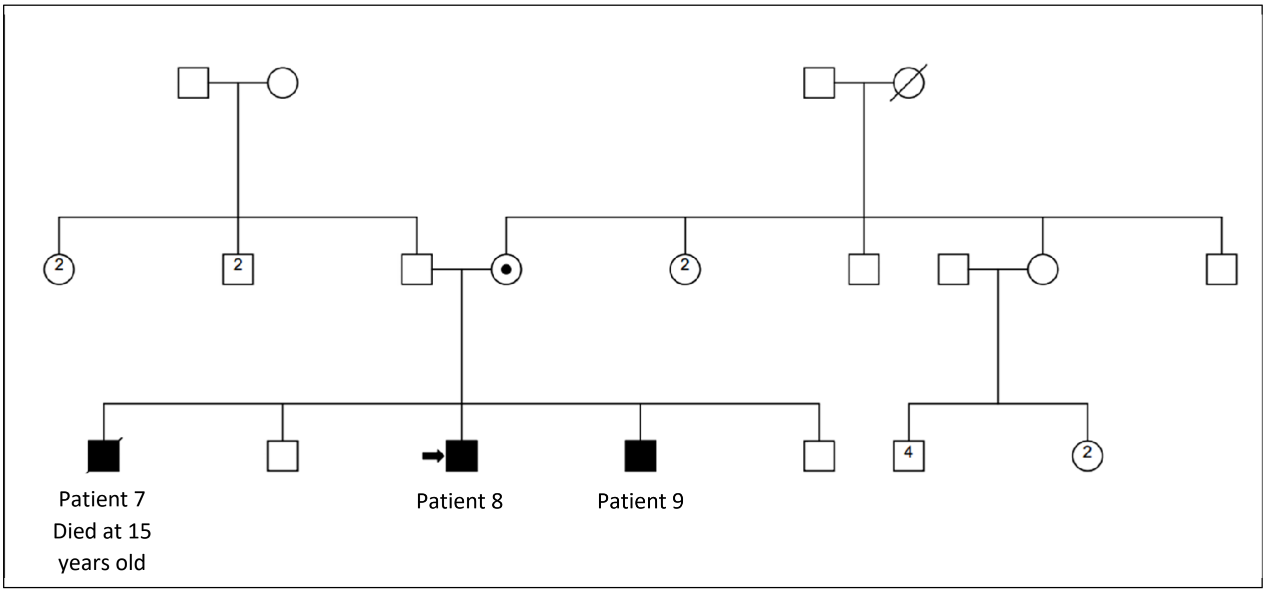

Figure S5: Pedigree of family 6

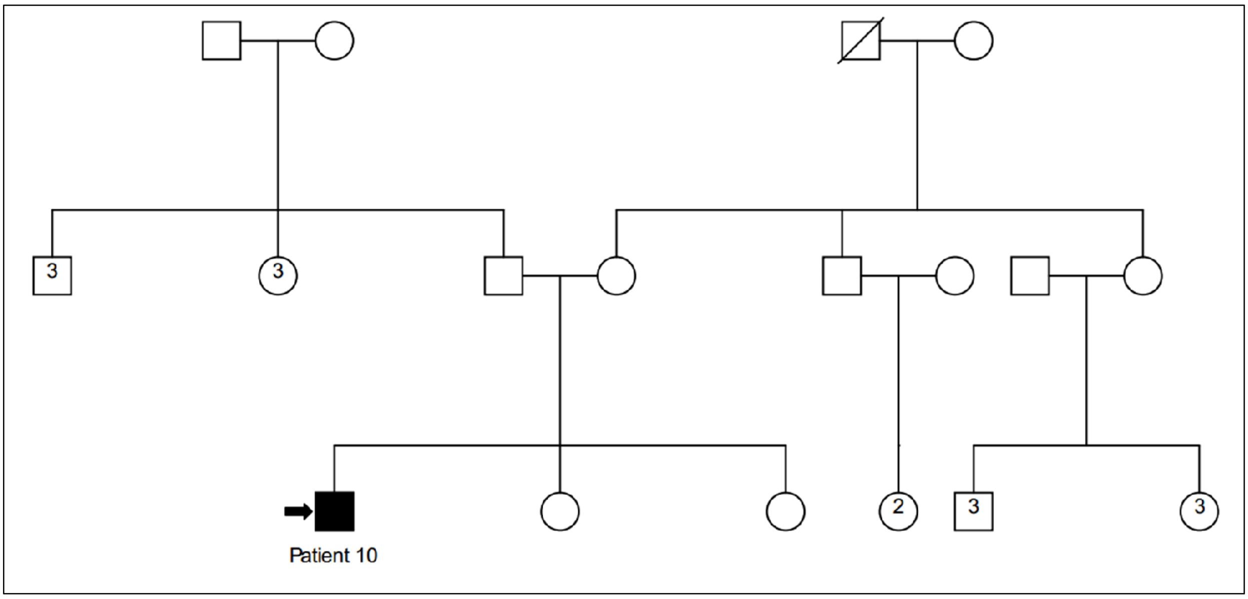

Figure S6: Pedigree of family 7

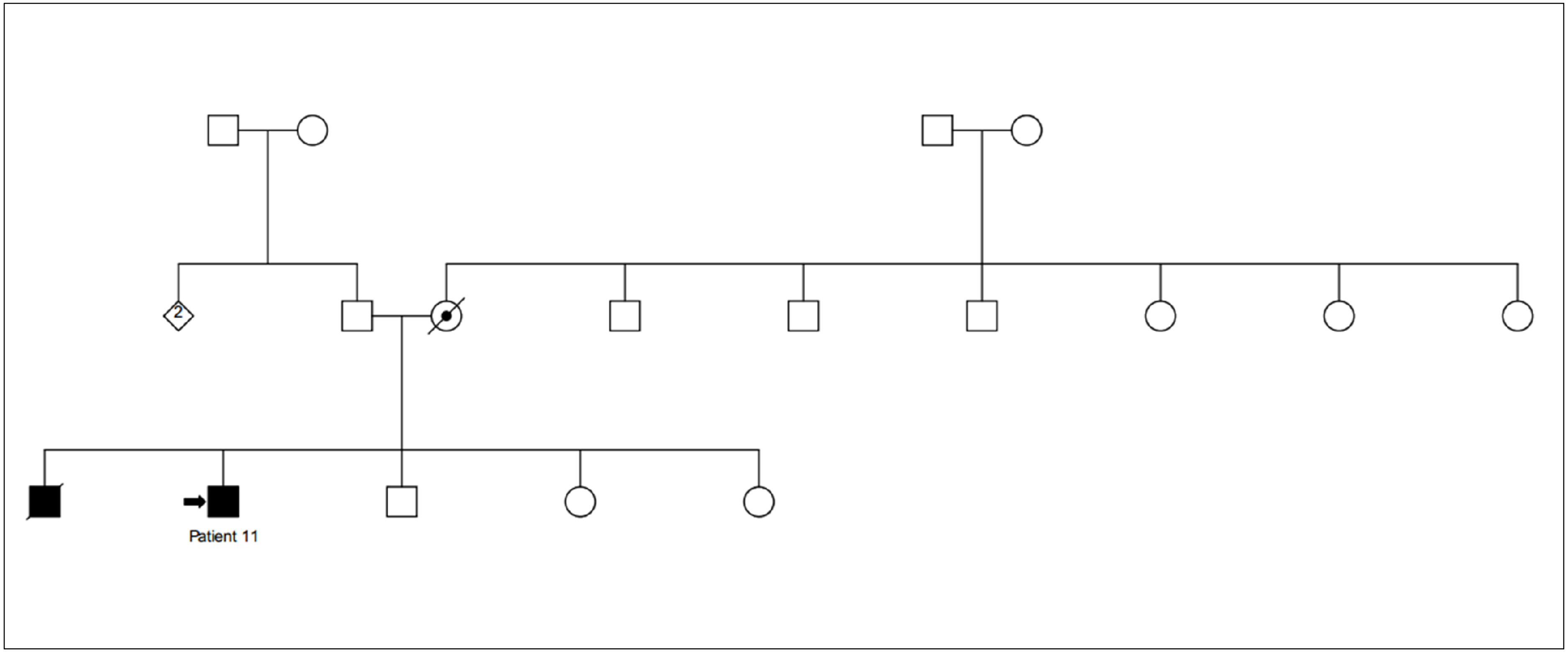

Figure S7: Pedigree of family 8

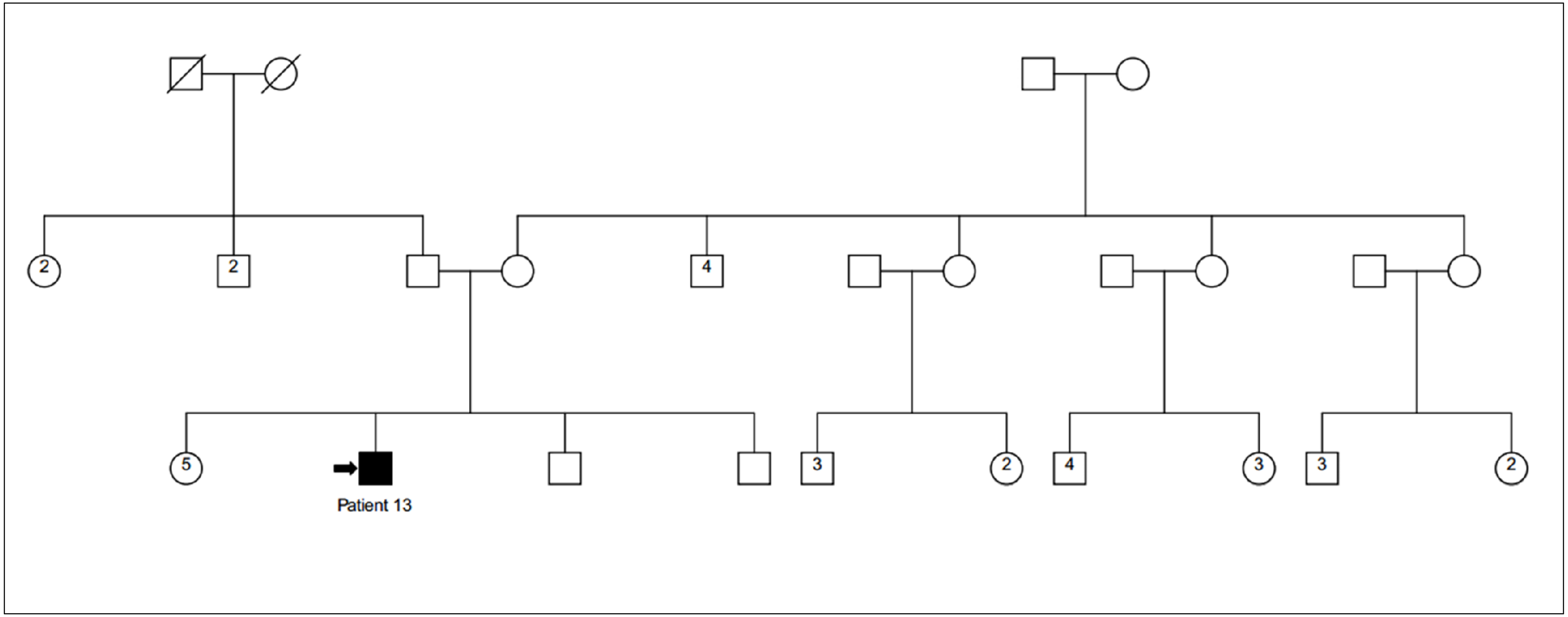

Figure S8: Pedigree of family 10

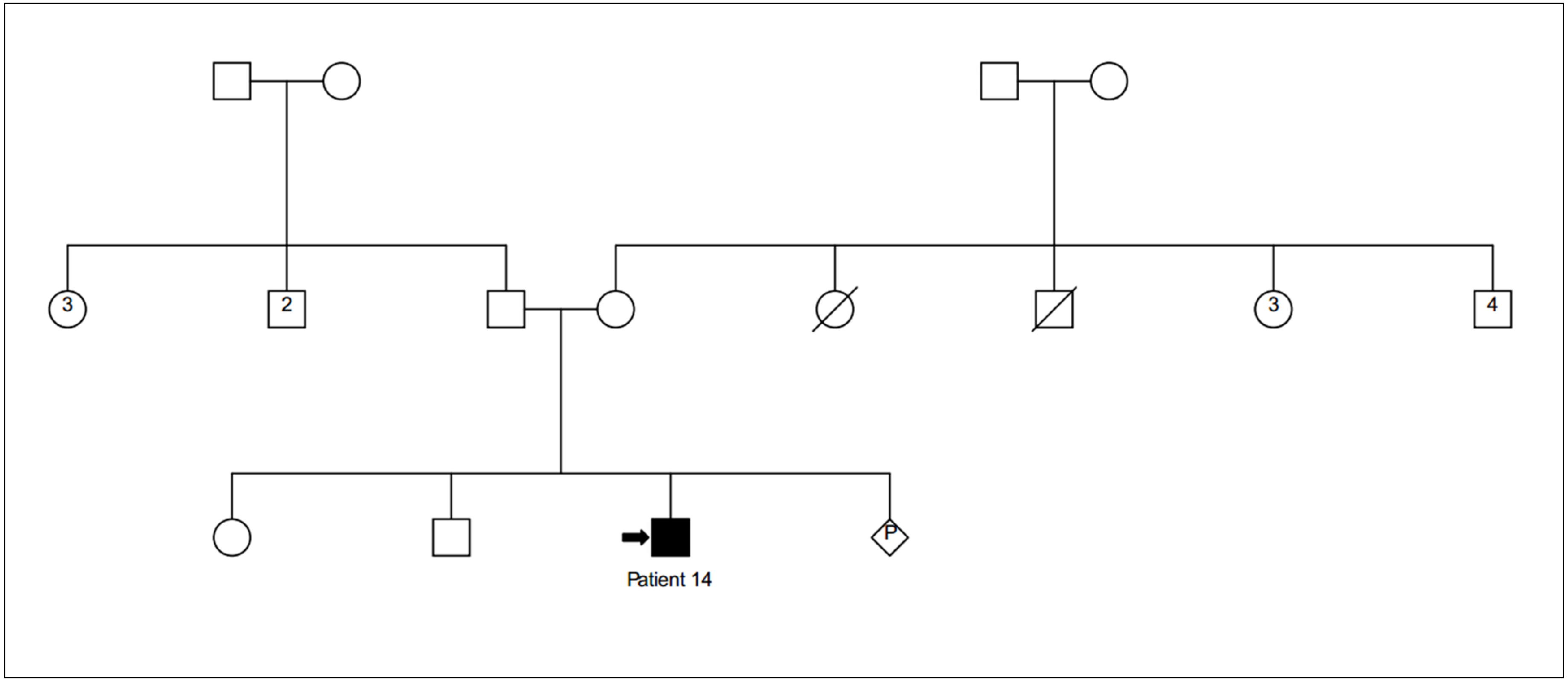

Figure S9: Pedigree of family 11

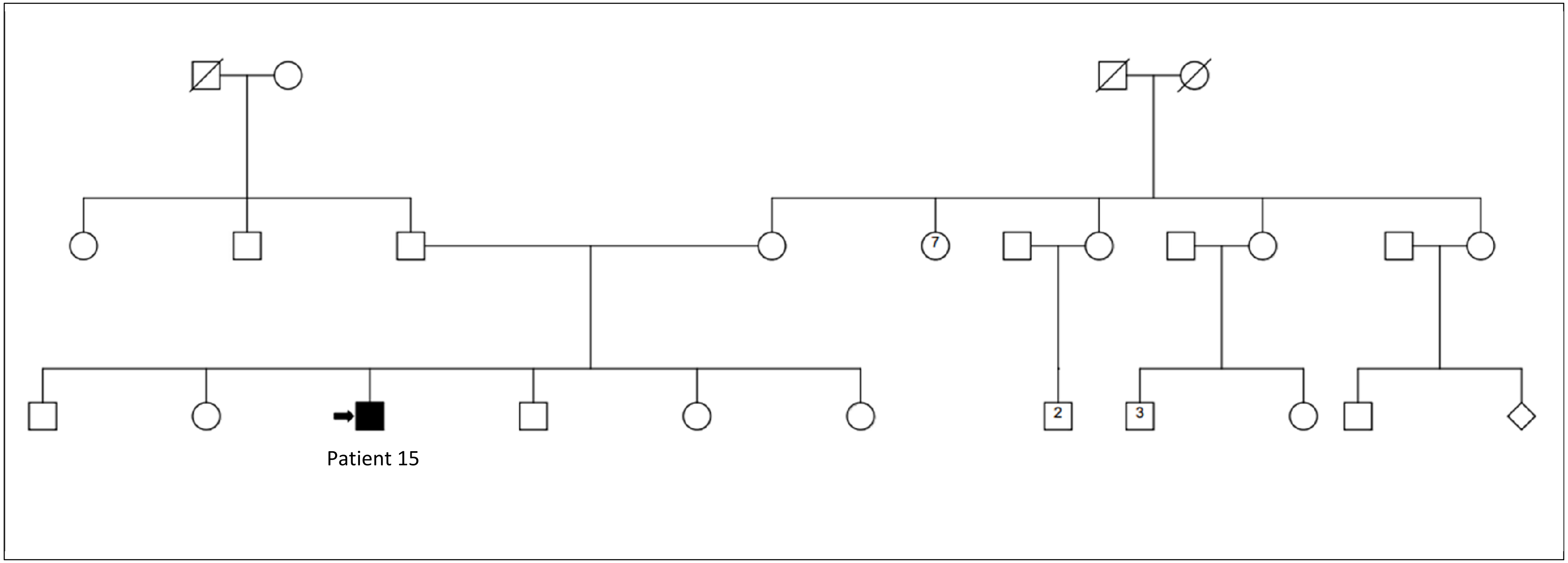

Figure S10: Pedigree of family 12

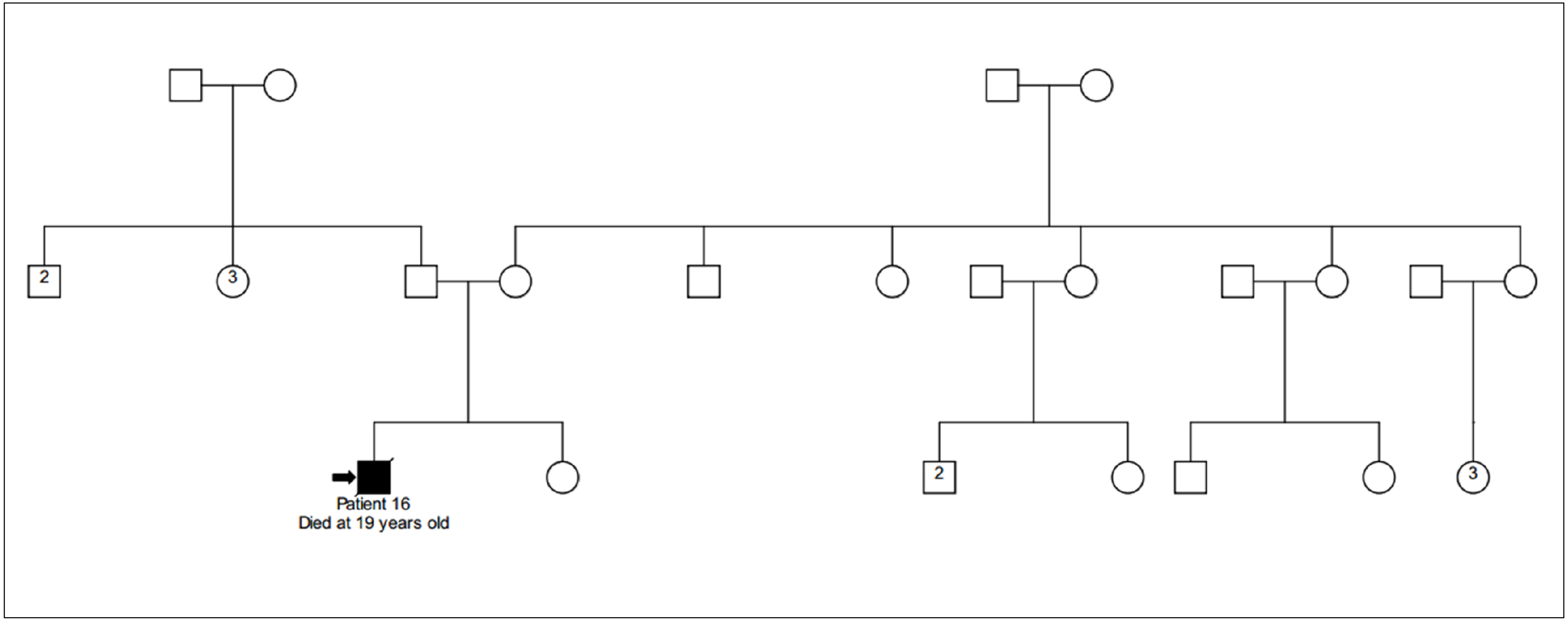

Figure S11: Pedigree of family 13

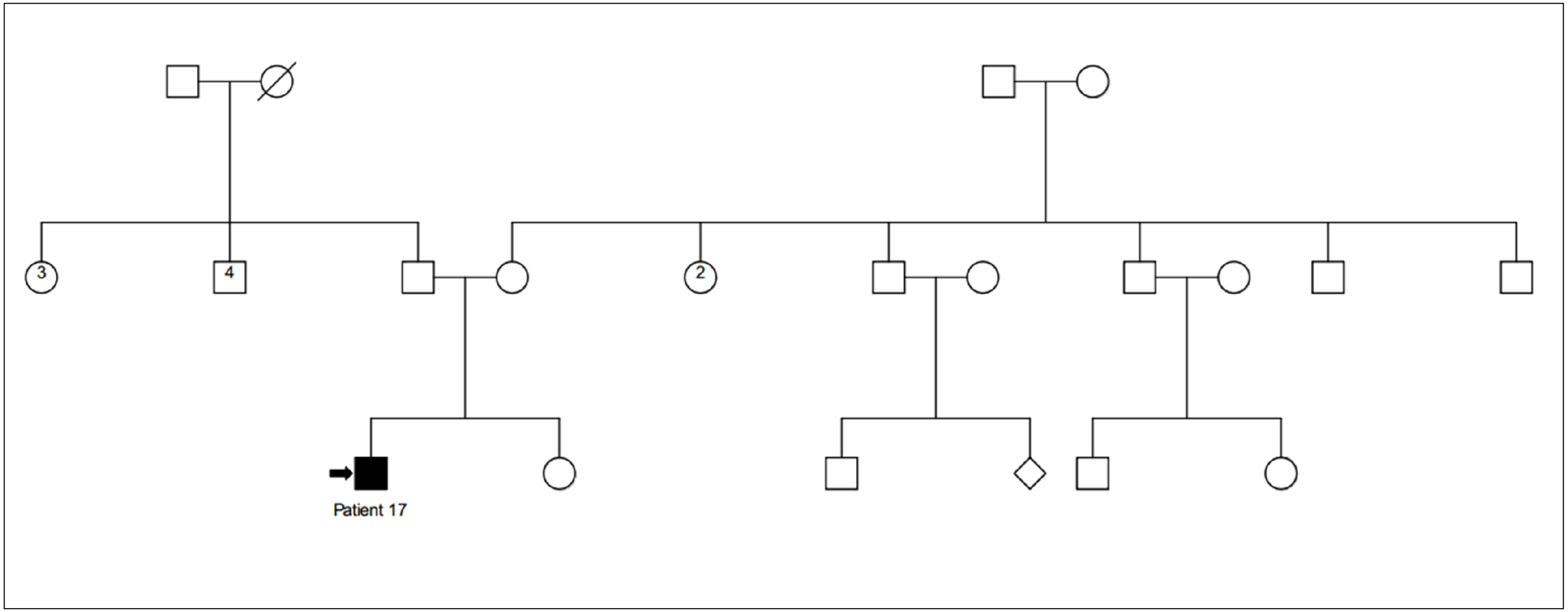

Figure S12: Pedigree of family 14
